# Bayesian Generalized Linear Mixed Modeling of Breast Cancer

**Published:** 2019-06

**Authors:** Ogunsakin ROPO EBENEZER, Siaka LOUGUE

**Affiliations:** Department of Statistics, School of Mathematics, Statistics, and Computer Science, University of Kwazulu Natal, Durban, South Africa

**Keywords:** Bayesian, Breast cancer, Multilevel, Generalized linear mixed modeling, CODA/BOA

## Abstract

**Background:**

Breast cancer is one of the most common cancers among women. Breast cancer treatment strategies in Nigeria need urgent strengthening to reduce mortality rate because of the disease. This study aimed to determine the relationship between the ages at diagnosis and established the prognostic factors of modality of treatment given to breast cancer patient in Nigeria.

**Methods:**

The data was collected for 247 women between years 2011–2015 who had breast cancer in two different hospitals in Ekiti State, Nigeria. Model estimation is based on Bayesian approach via Markov Chain Monte Carlo. A multilevel model based on generalized linear mixed model is used to estimate the random effect.

**Results:**

The mean age of the patients (at the time of diagnosis) was 42.2 yr with 52% of the women aged between 35–49 yr. The results of the two approaches are almost similar but preference is given to Bayesian because the approach is more robust than the frequentist. Significant factors of treatment modality are age, educational level and breast cancer type.

**Conclusion:**

Differences in socio-demographic factors such as educational level and age at diagnosis significantly influence the modality of breast cancer treatment in western Nigeria. The study suggests the use of Bayesian multilevel approach in analyzing breast cancer data for the practicality, flexibility and strength of the method.

## Introduction

Breast cancer refers to a malignant tumor developed from cells in the breast. Breast cancer is the most common cause of malignancy among women worldwide (1–3) and is a public health challenge among Nigeria women. Some years ago, breast cancer was not common in African countries especially Nigeria and was thought to be a leading course of death in the developed countries (4). Thirty Nigerian women die of breast cancer every day in 2008 and this had risen to 40 women in 2012. Over 508000 of Nigerian women died in 2011 as a result of breast cancer (5–7, 2). In a study about prevalence of breast cancer in western Nigeria found that breast cancer alone accounted for 37% of all the cancer cases present in western Nigeria (8).

With advancement in medical technology, advances have been made in the treatment of breast cancer among Nigeria women. Better treatment for breast cancer patients is difficult to define and older women are sometimes excluded from clinical treatment trials, probably because of their age (9). Since breast cancer biology differs from patient to patient with respect to factors like age, variation in response to treatment, and substantial competing risks of mortality (10–12), the exclusion of some patients might not be valid. This implies that those aged women included in trials are probably not a true representation for the general older population (13, 14). Because of this, treatment of cancer in women with concise strategy is urgently needed. Therefore, there is need for modeling of breast cancer using evidence-based strategy technique.

Prognostic factors for breast cancer especially in Western Nigerian had been well studied; there is paucity of data on population-based research. In addition, few studies have used Bayesian to establish the prognostic factors associated with the disease. Hence, we sought to determine risk factors for treatment given to female breast cancer in western Nigeria using generalized linear mixed models. This research focuses on the justification for the use of the conventional surgery method in the treatment of cancer, based on data from two understudied hospitals in western part of Nigeria, one federally owned, and the other state-owned. Hence, we aimed to provide knowledge on risk factors for breast cancer treatment modality, using both the classical and Bayesian approach via generalized linear mixed model.

## Materials and Methods

### Ethics

Ethical clearance to conduct the study was sought from the Ethical Review Committee of the Federal Teaching Hospital, Nigeria.

### Generalized Linear Mixed Model

Generalized linear mixed models (GLMMs) are an extension of linear mixed models to allow response variables from different distributions, such as binary responses. Alternatively, GLMMs is an extension of generalized linear models (e.g., logistic regression) to include both fixed and random effects (hence mixed models). The general form of the generalized linear mixed model is expressed:

[1] $$E(\varphi_{i}|\tau_{i},B,\vartheta_{k})=\tau_{i}^{\prime }\lambda +B^{\prime }\vartheta_{k}+\text{ò}$$

where *φ* is an *N_obs_* × 1 column vector, the outcome variable; *τ′_i_* is a N × p matrix of the p predictor variables; *λ* is a p × 1 column vector of the fixed-effects regression coefficients; B is an *N_obs_* × N*_r_* design matrix for the random effects (the random complement to the fixed *τ*); *ϑ_k_* is a q × 1 vector of the random effects (the random complement to the fixed *λ*); and is a *N* × 1 column vector of the errors.

The present study deals with a specific case of generalized linear mixed model where the response variable is binary and two levels of analyses (hospitals) are considered which is the best model to apply when we have this type of situation. Only hospital is considered at two levels in the model, then the B component is removed from the model in equation (1). For a given observation *i* within a hospital *k*, the generalized linear mixed model excluding the error term becomes:

[2] $$E(\varphi_{i}|\tau_{i},\vartheta_{k})=\tau_{i}^{\prime }\lambda +\vartheta_{k}$$

where *ϑ_k_* is the random effect representing the influence of hospital *k* on its within observation. Hence, the model utilized a logistic link function

[3] $$\begin{array}{l} E(\varphi_{i})=logit(\pi_{i})=log(\frac{\pi_{i}}{1-\pi_{i}})\\ logit(\pi_{i})=log(\frac{pr(\varphi_{i}=1|\tau_{i},\vartheta_{k})}{1-pr(\varphi_{i}=1|\tau_{i},\vartheta_{k})}) \end{array}$$

From equation (3), it implies that *π_i_* = *E*(*φ_i_* = 1|*τ_i_*, *ϑ_k_*) = *pr*(*φ_i_* = 1|*τ_i_*, *ϑ_k_*). Therefore, the final model is now expressed as:

[4] $$\pi_{i}=pr(\varphi_{i}=1|\tau_{i},\vartheta_{k})=\frac{exp[\tau_{1}^{\prime }\lambda +\vartheta_{k}]}{1+exp[\tau_{i}^{\prime }\lambda +\vartheta_{k}]}$$

where *τ_i_* represents the predictors for an individual *i* in an hospital *k* which is categorical. *φ_i_* denote the response variable, *λ* is the vector of the fixed regression coefficients and *ϑ_k_* is the random effect component (14). The estimated values of the regression parameters that maximize the probability of obtaining the observed data in classical statistics (15, 17) can be obtained by maximum likelihood. Likelihood of *k* independent measurements, given vectors of parameters *θ* and explanatory (predictors) variables *τ_i_* is represented as (15):

[5] $$p(\varphi |\theta ,\tau )=\prod_{i=1}^{k}p(\varphi |\theta ,\tau )$$

In case of binary logistic regression where response (outcome) variable *φ_i_* = 1 or 0, the likelihood function is estimated as (15):

$$p(\varphi |\theta ,\tau )=\{\begin{array}{c} logit^{-1}(\omega_{i})\;for\;\varphi_{i}=1\\ 1-logit^{-1}(\omega_{i})\;for\;\varphi_{i}=0 \end{array}$$

The above expression can be expressed as follows

$$\begin{array}{l} p(\varphi |\theta ,\tau )=\prod_{i=1}^{k}{\left(logit^{-1}(\omega_{i})\right)}^{\varphi_{i}}{\left(1-logit^{-1}\left(\omega_{i}\right)\right)}^{1-\varphi_{i}}\\ =\prod_{i=1}^{k}\pi^{\varphi_{i}}{\left(1-\pi_{i}\right)}^{1-\varphi_{i}} \end{array}$$

The estimate of *λ* that maximizes the likelihood function can be computed using advanced calculus. This is achieved by taking the logarithm of the likelihood function:

[6] $$log(p(\varphi |\lambda ,\vartheta_{k},\tau ))=\sum_{i=1}^{k}\left[\varphi_{i}log(\pi_{i})+(1-\varphi_{i})log(1-\pi_{i})\right]$$

Hence, the confidence interval of the parameters is then computed using Wald test and is represented as

[7] $$\lambda_{j}+Z_{1-\frac{z}{2}}SE(\lambda_{J})$$

where *λ_j_* is fixed effect coefficient associated with *j^th^* covariate, *SE* is the estimate of the standard error while $Z_{1-\frac{z}{2}}SE$ is the critical value for a standard normal distribution?

The classical statistics fit the logistic regression by means of an iterative procedure like maximum likelihood. In many situations, as a result of the assumptions underlying this iterative procedure, the estimation in classical statistics may result in non-convergence. These shortcomings as a result of non-convergence can be addressed using Bayesian inference as an alternative approach. Markov chain Monte Carlo algorithm approach can be used to provide a very general recipe for estimating properties of complicated distributions in Bayesian statistics without any difficulties. There obviously remain, however, some challenges in concluding whether Bayesian methods perform better than classical statistics in modeling breast cancer data. In this paper, we compared the result of classical statistics and Bayesian method. As a requirement of the Bayesian approach, several diagnostics tests were performed to ensure convergence of the Markov chain Monte Carlo and the true reflection of the posterior distribution.

### Bayesian Method

The methodology employed in this paper describes the Bayesian inference with emphasis on the posterior distribution, prior distribution as well as likelihood function for the Bayesian logistic regression. In the context of Bayesian statistics, data are regarded as fixed and unknown parameters as random variables. Considering a given parameter *θ* and a set of observed data, the interest of Bayesian technique lies on the probability of the parameter *θ* given the set of data *π*, i.e.*p*(*θ*|*π*). The main object of interest is the posterior distribution of the unknown parameters given the data. Mathematically, this can be written as

[8] $$p(\theta |\pi )=\frac{p(\pi |\theta )p(\theta )}{p(\pi )}\propto p(\pi |\theta )p(\theta )$$

Equation 8 is known as the principle of Bayesian method. Hence, *p*(*π*) is the prior distribution while the likelihood *p*(*θ*|*π*) is represented as:

$$\begin{array}{l} p(\theta |\pi )=\prod_{i=1}^{k}{\left[\pi_{i}\right]}^{P_{i}}{\left[1-\pi_{i}\right]}^{1-P_{i}}\\ \prod_{i=1}^{k}(\pi )=\frac{e^{\lambda_{0}+\lambda_{1}\tau_{1}+\ldots ,+\lambda_{k}\tau_{k}}}{1+e^{\lambda_{0}+\lambda_{1}\tau_{1}+\ldots ,+\lambda_{k}\tau_{k}}} \end{array}$$

While

$$1-\prod_{i=1}^{k}(\pi )=\left[1-\frac{e^{\lambda_{0}+\lambda_{1}\tau_{1}+\ldots ,+\lambda_{k}\tau_{k}}}{1+e^{\lambda_{0}+\lambda_{1}\tau_{1}+\ldots ,+\lambda_{k}\tau_{k}}}\right]$$

Hence, the likelihood function is expressed:

[10] $$p(\theta |\pi )=\prod_{i=1}^{k}{\left[\frac{e^{\lambda_{0}+\lambda_{1}\tau_{1}+\ldots ,+\lambda_{k}\tau_{k}}}{1+e^{\lambda_{0}+\lambda_{1}\tau_{1}+\ldots ,+\lambda_{k}\tau_{k}}}\right]}^{P_{i}}{\left[1-\frac{e^{\lambda_{0}+\lambda_{1}\tau_{1}+\ldots ,+\lambda_{k}\tau_{k}}}{1+e^{\lambda_{0}+\lambda_{1}\tau_{1}+\ldots ,+\lambda_{k}\tau_{k}}}\right]}^{1-P_{i}}$$

The preferred prior for logistic regression parameters is a multivariate normal distribution and is given as (15, 17, 18, 20):

[11] $$\lambda_{k}∼N(\varpi_{i},\sigma_{k}^{2})$$

Hence, we have:

$$p(\lambda_{k})\propto exp\left[-\frac{1}{2}{\left(\lambda_{k}-\varpi_{k})\right)}^{\prime }\sigma_{k}^{-1}\left(\lambda_{k}-\varpi_{k})\right)\right].$$

From equation [8], replacing *θ* with *λ_k_* result in *p*(*λ_k_*|*π*) ∝ *p*(*π*|*λ_k_*) *p*(*λ_k_*). Therefore, the posterior distribution for logistic regression (fixed effects only) is represented as:

[12] $$p(\lambda_{k}|\pi )\propto {\left[\frac{e^{\lambda_{0}+\lambda_{1}\tau_{1}+\ldots ,+\lambda_{k}\tau_{k}}}{1+e^{\lambda_{0}+\lambda_{1}\tau_{1}+\ldots ,+\lambda_{k}\tau_{k}}}\right]}^{P_{i}}{\left[1-\frac{e^{\lambda_{0}+\lambda_{1}\tau_{1}+\ldots ,+\beta_{k}\tau_{k}}}{1+e^{\lambda_{0}+\lambda_{1}\tau_{1}+\ldots ,+\lambda_{k}\tau_{k}}}\right]}^{1-P_{i}}\times exp\left[-\frac{1}{2}{\left(\lambda_{k}-\varpi_{k})\right)}^{\prime }\sigma_{k}^{-1}\left(\lambda_{k}-\varpi_{k})\right)\right]$$

Posterior distribution is usually of high dimension and analytically intractable which sometimes required knowledge of powerful integration. In order to overcome this complex nature of posterior, we employed Markov chain Monte Carlo (MCMC) algorithm. MCMC technique is among of the technique employed to generates the estimates of unknown parameters *θ* and corrects the values generated in order to have a better estimate of the desired posterior distribution (21, 31).

All the analysis in this study was carried out using WinBUGS (21). There were 1500000 iterations, with the first 5000 discarded to cater for the burn-in period. We assessed MCMC convergence of all models parameters by checking Gelman Rubin plot (17, 18). The scale reduction factor, also known as Gelman-Rubin convergence diagnostic (39) was calculated for model parameter to assess convergence and adequate mixing of the chains. The test statistics for the Gelman Rubin diagnostic test can be estimated as

$$\begin{array}{l} W\frac{1}{M}\sum_{m=1}^{M}S_{m}^{2}\\ B=\frac{n}{M-1}\sum_{m=1}^{M}{\left({\bar{\theta }}_{m}-\bar{\theta }\right)}^{2}\\ \hat{v}=\frac{n-1}{n}W+\frac{1}{n}B \end{array}$$

where *k* is the number of iterations of the chains.

$$\hat{R}=\frac{\hat{v}}{W}$$

Convergence is monitored when *R̂* → 1. *R̂* is called the estimated potential scale reduction factor.

## Results

### Socio-demographic characteristics of participants

Out of 237 patients, 192 cases accounting for (81.01%) were malignant breast lesions, while 45 cases (18.99%) were benign giving a ratio of 4.3:1 for malignant to benign breast lesion. The mean age of the respondents was 42.2 ± 16.6 yr with 52% of the women aged between 35–49 yr. 93.67% of those who participated in this study were Christians while 6.33% were Muslims. Various prognostic factors are considered which include: intercept (λ_0_), breast cancer types: malignant (λ_1_), age: 35–49(λ_2_), 50–69(λ_3_), 70+ (λ_4_), level of education: at least high school (λ_5_), religion: Christian (λ_6_), tribe: Yoruba (λ_7_), occupation: site of the cancer (λ_8_), hospital (λ_9_).

Table 1 gives a view of the sampled data with the following attributes: type of breast cancer, age, educational status, race, ethnicity, hospital and site of the breast cancer. The association between the variables of interest and the treatment of breast cancer patients received was calculated for the entire study (Table 1 model 3). Breast cancer (malignant) type was associated with the type of treatment given to patients, while other socio-economic factors were not significantly related, (model 3). The treatment given to breast cancer patients should be first based on the diagnosis of the breast cancer type. Hospital type was significant when breast cancer type was not included in the model (model 2). Hospital was significant but it’s influence passes through the type of breast cancer a patients was diagnosed. We can then say that the hospital a patient attended after being diagnosed with breast cancer played an important role in their chance of survival. Model 1 in Table 1 shows that the age and the hospital type were both significant with respect to the modality of treatment given to patient. The exclusion of type of breast cancer and educational status as a determinant for patients’ possibility of receiving surgical treatment makes age and hospital to be significant (model 1).

**Table 1:** Classical logit model for independent variables

***Parameter***	***Model M_3_ Lambda***	***Exact P-value***	***Model M_2_ Lambda***	***Exact P-value***	***Model M_1_ Lambda***	***Exact P-value***
λ_0_	−0.47[−5.83, 4.88]	1.000	3.96[1.67, 7.97]	0.011	3.95[1.66, 7.95]	1.000
λ_1_	-	-	-	-	5.01[3.06, 8.80]	<.001
λ_2_	−3.04[−8.17, 0.37]	0.118	−2.13[−6.12, 0.21]	0.088	−2.09[−5.95, 0.02]	0.040
λ_3_	−2.33[−7.43, 1.03]	0.375	−1.54[−5.49, 0.75]	0.299	−1.49[−5.40, 0.69]	0.290
λ_4_	−2.02[−7.09, 1.28]	0.528	−1.14[−5.05, 1.08]	0.546	−1.14[−5.05, 1.08]	0.546
λ_5_	0.36[−0.89,1.58]	0.698	0.09[−0.94, 1.11]	1.000	-	-
λ_6_	−0.50[−2.27, 1.67]	0.817	−0.45[−1.86, 0.91]	0.661	−0.45[−1.86, 0.92]	0.666
λ_7_	−0.72[−3.32, 2.66]	0.916	−1.24[−3.42, 0.84]	0.313	−1.25[−3.44, 0.85]	0.315
λ_8_	0.39[−1.80, 0.82]	0.694	−0.48[−1.42, 0.43]	0.360	−0.47[−1.42, 0.43]	0.367
λ_9_	0.48[−1.53, 0.60]	0.450	−1.99[−2.79, −1.24]	< 0,001	−1.98[−2.76, −1.26]	0.0001

Table 2 is the results for the model with Bayesian non-informative prior. From the 95% posterior intervals of λ*_j_*, we observe that only the posterior distribution of the age and malignant are away from zero, indicating a significant effect of these variables on the modality of treatment patients received from this part of Nigeria while other predictors considered in this study are not significant.

**Table 2:** Posterior Summary for Bayesian multilevel approach with non-informative

***Variable***	***Mean***	***SD***	***MC error***	***2.5%***	***Median***	***97.50%***
λ_0_	−0.217	2.314	0.033	−4.183	−0.288	4.127
λ_1_	−0.323	0.917	0.003	0.008	−0.367	1.611
λ_2_	0.672	0.556	0.002	−0.389	0.660	1.791
λ_3_	0.750	0.588	0.002	−0.365	0.736	1.949
λ_4_	3.735	1.988	0.007	0.561	3.495	8.237
λ_5_	0.680	1.348	0.007	−2.232	0.766	3.103
λ_6_	−6.417	1.510	0.005	−10.010	−6.195	−4.123
λ_7_	0.280	0.634	0.002	−0.894	0.255	1.590
*τ*.*Hosp*	3.375	5.095	0.021	0.012	1.505	17.830

### Assessing the performance of Markov Chain Monte Carlo (MCMC) chains in WinBUGS

**Fig. 1:**
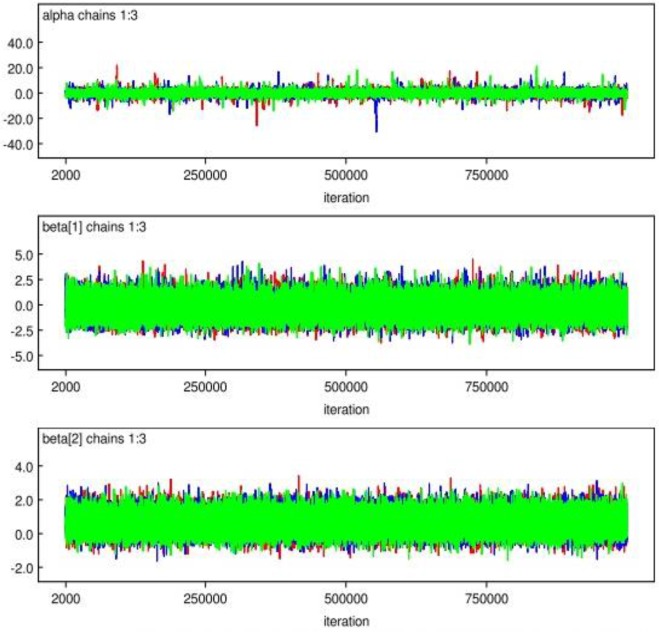
Convergence history for major independent parameters

**Fig. 2:**
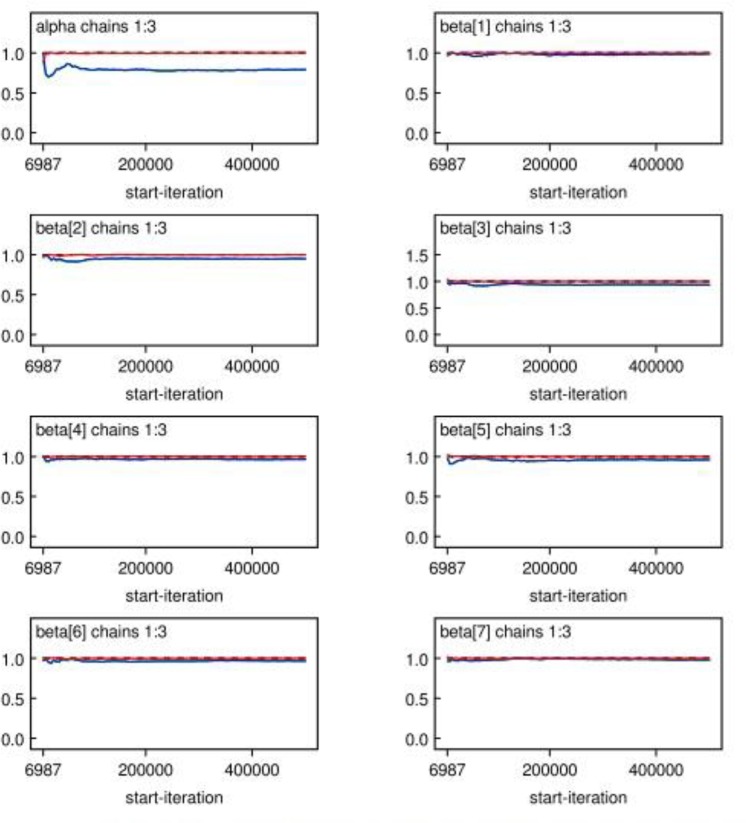
Gelman-Rubin statistics for major independent parameters

The performance of a diagnostic test can be examined in several ways. The diagnostics examined in this paper are those of Geweke (15), Heidelberger and Welch (16), and Raftery and Lewis (17), which look at convergence of an individual chain, and that of Gelman and Rubin (18), which bases convergence on analysis of multiple chains. In order to assess whether a chain has converged or not, we plot the sampled value against its number in the chain. Figure 1 displays the representation of the parameter behavior after 1,500,000 Monte Carlo repetitions. It was found that the kernel densities for shape and scale parameters exhibit approximately symmetric distribution. When the time series centered around a constant mean, it implies that the chain has reached convergence as reported in Fig. 2 and have the chains converged to the same solution.

## Discussion

Our results confirm findings from previous studies conducted in Nigeria on prognostic factors and breast cancer among women in Nigeria (1, 8, 2–13). Age could be a risk factor for breast cancer. Similar studies have been documented in other parts of Africa and the rest of the world (28, 29). Compared to the literature, the main contribution of the present study is the analysis of risk factors associated with modality of breast cancer treatment in western Nigeria in addition to the use of Bayesian approach. Previous studies that focus on the pattern of treatment given to breast cancer patients across time frame (years), age at diagnosis and educational categories are non-existent in western Nigeria. To the best of our knowledge, this could be the first study to address such scenario. For breast cancer treatment in Nigeria, the reason for surgery treatment on cancer patients is not clear (26). The present study showed prognosis factors for breast cancer treatment in western Nigeria.

In this study, patient with at least high school education is significant with treatment modality given to breast cancer, which is a new and unexpected finding in this part of Nigeria. From our analysis, the higher proportion of those treated with surgical treatment is found among educated and younger women. One of the possible explanations for these results may be that women with at least high school education presented their breast cancer cases to a medical practitioners than the less educated women. The result of Bayesian also reveals that those women with at least high school education are 2 times more likely to take surgery treatment than others. Other studies have shown similar result but not with treatment modality as investigated in the current finding (28–30, 32). We can infer from our findings that literacy level may influence treatment-seeking behavior. This finding could be used as an avenue to create awareness and advocacy campaigns that target less educated women in western Nigeria. Therefore, in the context of Nigeria, this is a good contribution in the area of breast cancer modeling as previous studies only found age at diagnosis as the risk factor that influence treatment pattern.

However, our study did not observe the influence of education on breast cancer patients, a recent study by (33) observed the impact of educational level on breast cancer risk using Swedish data. Women with higher education were more likely to be diagnosed with in situ breast cancer. In addition, patients who had low level of education were at higher risk of late presentation of their breast cancer (34, 35). An observation attributed to low level of awareness and knowledge about breast cancer treatment and screening.

Another finding from this study is that age at diagnosis is associated with treatment modality. Majority of the previous studies have indicated that age is an important prognostic factor for breast cancer screening but not many studies have examined age at diagnosis as a factor that influences the modality of breast cancer treatment in Nigeria particularly in western region. Therefore, this study adds to the large body of research indicating that age at diagnosis influence the pattern of treatment modality for breast cancer patients in Nigeria. Considering the effects of age in the treatment modality of breast cancer patient, Bayesian findings highlight that patients aged 35–49 yr are 0.67 times more likely to receive surgical treatment than their counterparts. This suggests that younger patients in western Nigeria were more likely to be treated with surgery, compared to the older patients, an observation supported by previous studies (3, 29, 30). Some studies attributed poor prognosis of breast cancer with age and the report indicated further that younger age is affected most due to an increase in invasive breast disease among this age group (3, 30, 32, 36). Our result also shows that malignant breast lesion was a predictor of breast cancer treatment and it appeared to have higher distribution among those who had at least high school education, an observation that supports previous studies (3, 37–42) that malignant breast lesion is predominant in this part of Nigeria.

## Conclusion

We have demonstrated the socio-demographic factors associated with treatment modality of breast cancer in western Nigeria. Since understanding the risk factors associated is necessary for curbing the menace of breast cancer prevalence, these results provide a rationale for the need to create more awareness campaigns, strategies and sensitization that target less educated women to enhance patronization of breast cancer screening in Nigeria as a whole.

## Ethical considerations

Ethical issues (Including plagiarism, informed consent, misconduct, data fabrication and/or falsification, double publication and/or submission, redundancy, etc.) have been completely observed by the authors.
